# Quadrantanopia as the only symptom of post-COVID stroke in the occipital pole: Case report

**DOI:** 10.1097/MD.0000000000027542

**Published:** 2021-11-05

**Authors:** Katarzyna Baltaziak, Agata Szpringer, Aleksandra Czarnek-Chudzik, Maksymilian Onyszkiewicz, Mario Damiano Toro, Anna Pankowska, Radoslaw Pietura, Robert Rejdak, Katarzyna Nowomiejska

**Affiliations:** a Chair and Department of General and Pediatric Ophthalmology, Medical University of Lublin, Poland; b Faculty of Medicine, Collegium Medicum Cardinal Stefan Wyszynski University, Warsaw, Poland; c Electroradiology Department, Medical University of Lublin, Poland.

**Keywords:** COVID-19, D-dimers, neuro-ophthalmology, quadrantanopia, SARS-CoV-2, stroke, thrombosis, visual field defect

## Abstract

**Rationale:**

This is a case report describing delayed complications of COVID-19 pneumonia, which evolved into the vascular-ischemic complications leading to quadrantanopia and MRI findings consistent with recent ischemic event in the occipital pole of the brain.

**Patient concerns:**

We report a case of a 46-year-old woman with quadrantanopia due to stroke confirmed with brain MRI, secondary to COVID-19 infection with chronically elevated D-dimers and treated with anticoagulation/antithrombotic modalities. Quadrantanopia was the only symptom recognized by the patient of a stroke localized in the occipital pole of the brain.

**Diagnosis:**

The patient was diagnosed with quadrantanopia due to stroke confirmed with brain MRI, secondary to COVID-19 infection.

**Intervention:**

Patient underwent ophthalmological examination and MRI.

**Outcomes:**

A thrombotic or ischemic risks in the chronic recovery from COVID-19 should be considered in patients with elevated D-dimers.

**Lessons:**

An MRI should be considered as a long term follow up for post-COVID-19 patients reporting ophthalmic or neurologic complains.

## 1 Introduction

In December 2019, a novel corona virus associated with a series of acute, atypical cluster of pneumonia cases and respiratory symptoms was first detected in Wuhan, a city in the Hubei Province of China. Since then, the virus now known as SARS-CoV-2 (severe acute respiratory syndrome coronavirus 2), has spread to over 200 countries and it continues to be recognized as a major world pandemic.^[1]^ Coronaviruses are a family of enveloped RNA viruses that are distributed widely among mammals and birds, causing principally respiratory or enteric disorders, and in some cases neurologic illness or hepatitis.^[2]^ SARS-CoV2 is known to initially bind to the angiotensinconverting enzyme 2 receptors of epithelial and endothelial cells where an immediate immunological activation occurs that can, in severe cases, eventually lead to hypercoagulability or thrombophilia and increased tendency of clots forming in the blood and potentially acute ischemic stroke (AIS).^[3]^ In addition, non-respiratory clinical manifestations of the CoV-related disease, mainly ophthalmological and neuro-ophthalmological were also reported but not fully described.^[4]^ For example conjunctivitis and keratoconjunctivitis^[5]^ as well as retinal alterations^[6]^ and diplopia and ophthalmoparesis^[7]^ have been described as case reports or case series. We believe that this was at least in part due to both technical and safety issues concerning the detailed ophthalmological examinations in CoV patients and the legitimate tendency to neglect eye complaints due to life-threatening manifestations taking priority over the secondary symptoms.^[8]^

It is assumed, that about one-third of patients hospitalized due to severe COVID-19 develops macrovascular thrombotic complications, including venous thromboembolism, myocardial injury/infarction, and stroke.^[9]^ Stroke is perhaps the most serious non-pulmonary complication of COVID-19.

In some patients, COVID-19 infection can be severe, with hypercoagulation being a common finding, with a vascular endothelial damage and the consequent risk of venous and arterial thrombotic complications. Coagulopathy secondary to COVID-19 infection significantly worsens the prognosis and increases overall mortality. Anosmia and dysgeusia have been reported as common early symptoms of COVID-19 in absence of nasal congestion or discharge, but severe neurologic presentations are rather rare with the exception of encephalopathy secondary to hypoxemia in the course of SARS-CoV-2 acute respiratory syndrome. Vascular and infectious encephalopathy had been correlated with COVID-19 in critically ill patients. However, whether COVID-19 may be considered a risk factor for stroke is still not established.^[10]^

There are many publications regarding broad range of respiratory-related COVID-19 manifestations, but there are very limited sources of data related to neuro-ophthalmologic complications of COVID-19.^[11]^ The aim of this study was to present a case of quadrantanopia as the only symptom of post-COVID stroke in the occipital pole.

## 2 Case description

A 46-year-old previously healthy woman without obesity (BMI = 23 kg/m^2^) and cardiovascular risk factors and no personal or family history of thromboembolic events reported fever, cough, and myalgia in January 2020 after returning from her trip to Andora. After computed tomography scan of the chest with extensive multifocal dense ground-glass opacities in the lungs she was diagnosed with pneumonia and was treated in the hospital with 3 antibiotics and antiviral medication. Her illness was lasting 4 weeks. She had no neurologic signs during infection. D-dimer levels were as follows: 882 ng/mL on February 3, 2020, 3412 ng/mL on February 8, 2020, and 421 ng/mL on February 13, 2020 and the anticoagulation treatment was reinitiated with Clexane (enoxaparin sodium) given subcutaneously. Her consecutive levels of D-dimers were not normalizing during the course of the illness regardless the continued treatment. Additional studies in the courses of the hospitalization included ultrasound of the abdomen, Doppler ultrasound of lower limbs’ venous system, Doppler ultrasound of cervical/carotid and vertebral arteries, and all showed no abnormalities. At that time, the patient did not undergo a polymerase chain reaction diagnostic test for SARS-CoV-2 due to the pre-pandemic onset of illness. The patient's daughter had developed similar symptoms and virus pneumonia after 2 weeks since the contact. According to the official data at that time there were no known cases of COVID-19 in Poland.

On the July 3, 2020, the patient had D-dimers tested again due to severe dizziness and the level was 1193 ng/mL, and she was treated with acetylsalicylic acid (75 mg). In September 2020 the D-dimers level was 600 ng/mL.

**Figure 1:**
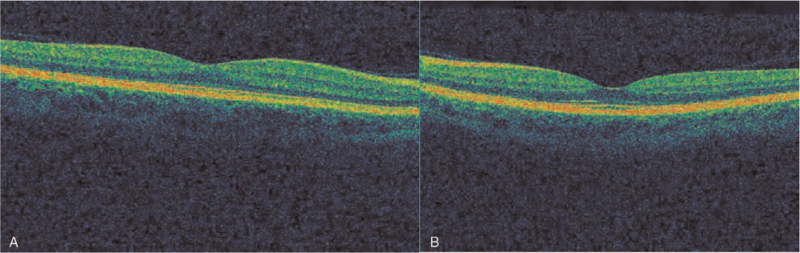
Optical coherence tomography of the macula of the right (A) and left (B) eye of the presented patient – normal appearance.

**Figure 2:**
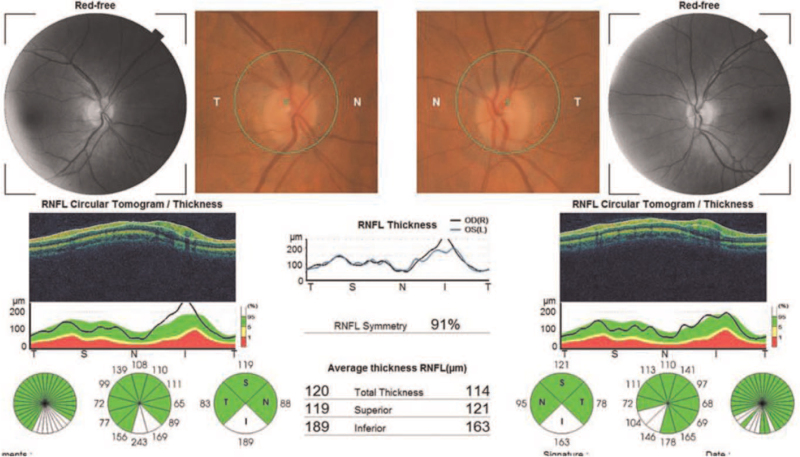
Optical coherence tomography of the peripapillary retinal nerve fiber layer thickness and photography of the optic disc of both eyes of presented patient – normal result.

**Figure 3:**
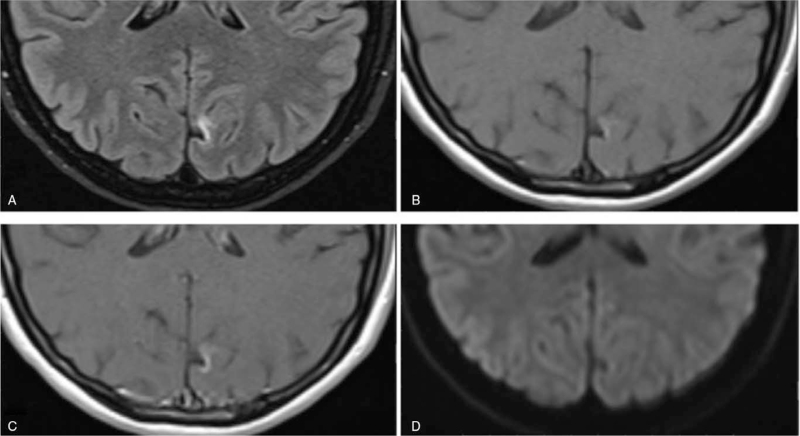
MRI study, axial brain slices of the occipital pole showing ischemic stroke obtained using following sequences: (A) Turbo inversion recovery magnitude (TIRM), (B) T1-weighted turbo spin echo (TSE), (C) T1-weighted TSE after gadolinium contrast administration, and (D) diffusion-weighted imaging (DWI).

In October 2020 the patient presented at the Department of General Ophthalmology in Lublin and the ophthalmological tests have been completed due to bilateral visual deterioration mentioned by the patient. Best-corrected visual acuity was 0.8 the right eye and 0.9 in the left eye. Anterior segment examination and intraocular pressure were not remarkable. Fundoscopic examination and photography was normal, optical coherence tomography of the macula and peripapillary retinal nerve fiber layer thickness – also normal (Figs. 1 and 2). There was no pain on ocular movements and there was no relative afferent pupillary defect. Based on the visual fields examination (semi-automated kinetic perimetry using Octopus 900) the quadrantanopia was diagnosed in lower and right area in both eyes (Fig. 3).

The patient was referred to neurologist for additional evaluation. The neurological evaluation revealed isolated right homonymous lower quadrantanopia, the National Institutes of Health Stroke Scale score was 1 point. Routine computed tomography scan of the head did not show any pathology.

The MRI examination was conducted using Magnetom-Avanto 1.5 T scanner (Siemens Healthcare, Erlangen, Germany). Study protocol comprised turbo inversion recovery magnitude sequence, diffusion-weighted imaging, and apparent diffusion coefficient maps, T1- and T2-weighted sequences with and without gadolinium enhancement obtained in coronal, sagittal, and axial planes. Within the medial cortex of the left occipital lobe, a banded high-signal area in turbo inversion recovery magnitude and T1-weighted sequence with the presence of cortical laminar necrosis areas and a slight enhancement after intravenous gadolinium administration was visible. Diffusion-weighted imaging/apparent diffusion coefficient imaging did not show diffusion restriction. The image obtained in MRI examination corresponds to the chronic vascular ischemic areas (Fig. 4).

Patient was admitted to the stroke unit of the neurological department to perform additional investigations. Duplex Doppler ultrasound assessing the cerebral arteries in the intracranial segments showed an occlusion of the left posterior cerebral artery.

In order to search for a cardioembolic origin of the embolus, additional cardiological diagnostic examinations were carried out. Echocardiography did not reveal any valvular defects or myocardial contractility disorders; 24-hour Holter ECG monitoring did not disclose significant arrhythmias or conduction disorders. Patient was monitored for blood pressure and blood sugar.

To determine the etiology of cerebral stroke additional laboratory tests towards rare blood diseases of increased coagulability states were carried out but without findings (homocysteine level, antiphospholipid antibodies, anticardiolipin antibodies, antinuclear antibodies, antineutrophil cytoplasmic antibodies, factor V Leiden mutation, protein C and S level, antithrombin III level).

Considering the age of the patient and the absence of serious concomitant cardiovascular diseases SARS-CoV-2 infection was taken to be an independent cerebrovascular risk factor.

As the patients arrived beyond the time of the therapeutic window for thrombolysis (4.5 hours) this intensive treatment of AIS was not taken. As the secondary prevention of stroke acetylsalicylic acid was given orally.

The patient was regularly followed-up by ophthalmologist and neurologist, but the visual acuity and neurological state were both stable. Visual acuity was 0.9 in both eyes after 6 months, the quadrantanopia was still present.

## 3 Discussion

To date, neurologic comorbidities and complications of COVID-19 infections were broadly described including dysosmia, dysgeusia, Guillain-Barre syndrome, anosmia, and AIS. Rarely, neuro-ophthalmology complications of COVID-19 have been reported.^[12,13]^

This is a case report describing complications of COVID-19 pneumonia, which evolved into the vascular-ischemic complications leading to quadrantanopia few months later and MRI findings consistent with recent ischemic or thrombotic event in the occipital pole of the brain. Neuroimaging abnormalities had been described in patients with COVID-19-related encephalopathy, stroke, encephalitis, and other complications. The incidence of ischemic stroke associated with COVID-19 in hospitalized patients has been under 3%^[14]^ and focal lesions leading to quadrantanopia has not been described, yet. Reviews of COVID-19 publications, reported clinically and autopsy-determined rates of acute brain infarction range from 0.4% to 8.1% while rates of acute brain hemorrhage range from 0.13% to 9.5%.^[10]^ Based on large meta-analysis, 1.4% of patients with COVID-19 suffer from cerebrovascular disease.^[10]^

It has already been observed that patients with COVID-19 and stroke are younger than patients with stroke without infection^[15]^ and majority of post-COVID patients (87.4%) develop AIS rather than intracerebral hemorrhage (11.6%).^[10]^ Moreover, people with COVID-19 developing a stroke were older than infected patients without stroke.^[10]^ None of the studies reported visual field results in patients with stroke. In a study of Mao et al, visual symptoms, not specified, were found in 4 of 214 patients with neurological symptoms accompanying COVID-19 infection. In our case report of post-Covid stroke we have shown documentation with the peripheral visual field results and localization of ischemic area in the occipital pole of the brain.

A recent vascular/thrombotic event on MRI in studied patient was consistent with AISs in patients with COVID-19 reported in several case series and several mechanisms for AIS in COVID-19 were proposed including hypercoagulability, vasculitis, new onset atrial fibrillation, and the infection by the SARS-CoV-2 itself.^[16]^

Connors and Levy,^[17]^ recommended that D-dimer, PT, aPTT, fibrinogen, and platelet count testing could be prognostic at admission. A coagulation panel is not typically ordered in ophthalmology services, but we are convinced now that the rising D-dimer and the rapid drop in fibrinogen and platelet count could be associated with neuro-ophthalmologic comorbidities leading to complications in vision impairment or loss. In COVID-19, D-dimer elevation appears to be an important prognostic marker and has been observed in initial COVID-19 stroke patients, as in our patient.

The American Stroke Association has indicated that the risk of stroke doubles every 10 years after age 55, so far more elderly people are affected by stroke.^[18]^

Due to the increase in COVID-19 infections, the literature reports an increasing number of premature strokes also in the younger generation.^[19]^

Early reports from China described cerebrovascular disease in about 5% of patients with severe COVID-19 disease, with strokes occurring almost 2 weeks after initial diagnosis. In our study, the stroke occurred probably few weeks after COVID-19 infection, the precise moment is not known, as the neurological signs were limited to quadrantanopia.

Reports on visual disturbances during or post-COVID infection are scarce^[14]^ and not described in details, only as accompanying neurological disturbances.

Recently developed treatments, such as intravenous thrombolysis and mechanical thrombectomy, can significantly improve the outcomes of AIS. However, the effects of these treatments are highly time-dependent. After the end of a therapeutic time window (4.5 hours) according to recommendations we can use antiplatelet drugs, hypotensive drugs and statins. In our case it is not possible to determine precisely when the thromboembolic event occurred. The present case of stroke delayed after COVID-19-related pneumonia seems to confirm that COVID-19 is an independent risk factor for AIS.

In addition to the potential ischemic event, we have considered thrombotic risks and we have used anticoagulating and antithrombotic therapy (low-molecular-weight-heparins) in responses to elevated D-dimers keeping in mind a prevention of the acute visual loss due to ophthalmic artery occlusion. Fortunately, it was a low risk patient since the carotid and intracerebral circulation was normal based on the U/S and CTA, respectively.

Nevertheless, the optimal treatment to prevent ischemic event (ischemic stroke) or vascular thrombosis in presence of D-dimer elevation in COVID-19 neuro-ophthalmic complications continue to be unclear and require attention to multimodalities in diagnostic evaluations and multispecialty team's collaboration in diagnostic evaluations of such patients.

## Author contributions

**Conceptualization:** Mario Damiano Toro.

**Funding acquisition:** Robert Rejdak.

**Methodology:** Maksymilian Onyszkiewicz.

**Resources:** Anna Pankowska, Radoslaw Pietura.

**Visualization:** Agata Szpringer, Aleksandra Czarnek-Chudzik.

**Writing – original draft:** Katarzyna Baltaziak.

**Writing – review & editing:** Katarzyna Nowomiejska.
